# Histology-Validated Dielectric Characterisation of Lung Carcinoma Tissue for Microwave Thermal Ablation Applications

**DOI:** 10.3390/cancers15143738

**Published:** 2023-07-23

**Authors:** Laura Farina, Giuseppe Ruvio, Ramadan Shatwan, Aliaa Shalaby, Martin O’Halloran, Alexandra White, Alan Soo, David Breen, Aoife Lowery, Anne Marie Quinn

**Affiliations:** 1 R & D, Endowave Ltd., H91 DCH9 Galway, Ireland; giuseppe@endowave.ie; 2 College of Medicine, Nursing and Health Sciences, University of Galway, H91 TK33 Galway, Ireland; martin.ohalloran@universityofgalway.ie; 3 Department of Anatomic Pathology, Galway University Hospital, H91 YR71 Galway, Ireland; ramadan.shatwan@hse.ie (R.S.); aliaa.shalaby@mailn.hse.ie (A.S.); annema.quinn@hse.ie (A.M.Q.); 4 Department of Cardiothoracic Surgery, Galway University Hospital, H91 YR71 Galway, Ireland; alexandrawhite@rcsi.ie (A.W.); alan.soo@hse.ie (A.S.); 5 Interventional Respiratory Unit, Department Respiratory Medicine, Galway University Hospital, H91 YR71 Galway, Ireland; david.breen@hse.ie; 6 Discipline of Surgery, School of Medicine, University of Galway, H91 TK33 Galway, Ireland; aoife.lowery@universityofgalway.ie

**Keywords:** transbronchial microwave ablation, dielectric properties, non–small cell carcinoma

## Abstract

**Simple Summary:**

Our study provides the first accurate dielectric characterisation of human lung tumours and parenchyma. Data were collected from neoplastic and non-neoplastic pulmonary tissue in the microwave frequency range prior to scheduled pathology analysis. On average, values from pulmonary tumours were found to be twice the value of normal lung parenchyma. Therefore, tumours will be more apt than the surrounding parenchyma to absorb the microwave energy radiated by a transbronchial microwave applicator.

**Abstract:**

Microwave thermal ablation is a promising emerging treatment for early-stage lung cancer. Applicator design optimisation and treatment planning rely on accurate knowledge of dielectric tissue properties. Limited dielectric data are available in the literature for human lung tissue and pulmonary tumours. In this work, neoplastic and non-neoplastic lung dielectric properties are characterised and correlated with gross and histological morphology. Fifty-six surgical specimens were obtained from twelve patients undergoing lung resection for lung cancer in University Hospital of Galway, Ireland. Dielectric spectroscopy in the microwave frequency range (500 MHz–8.5 GHz) was performed on the ex vivo lung specimens with the open-ended coaxial probe technique (in the Department of Pathology). Dielectric data were analysed and correlated with the tissue histology. The dielectric properties of twelve lung tumours (67% non-small cell carcinoma (NSCC)) and uninvolved lung parenchyma were obtained. The values obtained from the neoplastic lung specimens (relative permittivity: 52.0 ± 5.4, effective conductivity: 1.9 ± 0.2 S/m, at 2.45 GHz) were on average twice the value of the non-neoplastic lung specimens (relative permittivity: 28.3 ± 6.7, effective conductivity: 1.0 ± 0.3 S/m, at 2.45 GHz). Dense fibrosis was comparable with tumour tissue (relative permittivity 49.3 ± 4.6, effective conductivity: 1.8 ± 0.1 S/m, at 2.45 GHz).

## 1. Introduction

Lung cancer is the leading cause of cancer-related deaths worldwide. The majority of patients are diagnosed with advanced-stage disease at the time of initial presentation. Despite recent advances in surgery, chemotherapy and radiotherapy, the mortality associated with lung cancer remains high, with a five-year mortality rate over 80%. Data from screening trials have shown that earlier-stage cancers are associated with better outcomes, with substantially higher five-year survival rates [1].

Advances in image-guided techniques [2,3], low-dose computed tomography (CT) scans for screening [4] and robotic platforms for lung navigation [5] are providing more reliable access of diagnostic tools into pulmonary parenchymal lesions [6,7]. This, in turn, creates a safe and more accurate path to the target tumour, which, in turn, can be used for minimally invasive treatments such as radio-frequency, cryo- and microwave ablation. Transbronchial ablative treatments have now the potential to mitigate risks associated with a percutaneous approach and increase the spectrum of treatment options for lung cancer patients, especially those who may not be candidates for surgical management [4,7].

Microwave ablation is emerging as a promising novel minimally invasive therapeutic option. It has improved energy delivery capability compared with radio-frequency ablation [4,8], thus making it a suitable technique for a transbronchial approach to tumour treatment [7,9,10]. The use of microwave ablation in the lung is currently under investigation in both pre-clinical and clinical studies; however, limited research has been undertaken to characterise the properties of human lung tissue [11,12]. The knowledge base concerning the electromagnetic response of healthy and diseased lung tissue is minimal compared to the number of investigations conducted on the liver [13,14,15], where microwave ablation has demonstrated clinical success [16,17].

Intrinsic dielectric properties, namely, relative permittivity and effective conductivity, have a fundamental role in microwave energy distribution and absorption and impact the final ablated volume. Relative permittivity measures the tissue’s ability to accept an electric field, such as that radiated by a microwave ablation applicator [18]. Relative permittivity dictates the energy propagation through the tissue and the most appropriate radiating antenna design. Higher permittivity facilitates controlled energy propagation and more minimally invasive applicator designs. Effective conductivity measures the tissue’s ability to absorb microwave energy [18]. Higher conductivity is linked to higher water content and poorer air content. Higher conductivity increases microwave energy absorption efficiency, thus also resulting in higher heating efficiency.

The primary endpoint of this study is to provide a comprehensive investigation of the dielectric properties of normal lung tissue and benign and malignant lung specimens. The dielectric properties enable the optimisation of controlled energy transfer from microwave ablation devices to treat lung cancer, and thereby provide guidance for the development, optimisation and assessment of medical devices with diagnostic and/or therapeutic purpose based on the interaction of an electromagnetic field with lung tissue.

This study presents:

1. The measurement results of dielectric property values (permittivity/conductivity) across the microwave frequency range between 500 MHz and 8.5 GHz for each tissue sample with associated deviation range;
2. The dielectric property values of each tissue group (lung parenchyma based on its clinical condition and lung tumours based on their pathology classification) and the associated correlation with the intrinsic and pathological tissue characteristics.

The measurable outcomes of the study are the dielectric properties of each lung sample and tissue group.

## 2. Materials and Methods

### 2.1. Patients and Tissue Specimens

In total, 12 patients with lung cancer were enrolled in the study in University Hospital Galway, Ireland, over a period extending from October 2020 to July 2021. Overall, fifty-six specimens were obtained from twelve lung resections. Prior to resection, the specimens to be sampled were identified by the thoracic surgeon and pathologist based on tumour location, size (10 mm or more) and accessibility. Patients with a recent or current diagnosis of COVID-19 infection were excluded from the study in order to minimise the risk of aerosol transmission to the clinical and pathology team members, as the study involved fresh tissue analysis.

The study was approved by the NUI Galway Research Ethics Committee, reference number C.A. 2447, and conducted in the Histopathology Laboratory (Department of Anatomic Pathology) of University Hospital Galway (UHG), Ireland. Patient consent was obtained before proceeding.

Lung tissue was analysed in a fresh state for dielectric properties within a ventilated fume hood. Tissue blocks of the measured areas were then fixed in 10% neutral-buffered formalin within 3 h of resection. Tumour and normal lung tissue morphology (based on a haematoxylin and eosin stain with additional typing information based on immunohistochemistry) was reviewed by three pathologists (A.M.Q., R.S., A.S.).

The tissue surface areas ranged from 100 to 300 mm^2^.

### 2.2. Dielectric Spectroscopy

A total of 112 measurement points were identified and acquired from 56 tissue blocks taken from 12 lung resection specimens. Overall, 56 histological cassettes were processed, each containing at least one measurement point. On final analysis, 49 measurement points were identified as cancerous and 63 as non-cancerous tissue.

Dielectric property measurements were performed following the current standard of practice in the field [13,14,19,20,21]. The open-ended coaxial probe (OECP) technique was adopted. A slim-form probe (Keysight 85070E, Santa Rosa, CA, USA) with a 2.2 mm diameter, 1 mm sensing depth and 3 mm sensing radius (1 mm beside the diameter of the probe) [22,23] was used. This probe dimension allows consistent contact with the tissue sample, resulting in robust performance. The probe was connected to a vector network analyser (Keysight VNA E5063A, Santa Rosa, CA, USA) through a low-loss coaxial cable, to acquire and process the reflection coefficient data. The data were recorded at 101 linearly spaced frequency points over a frequency range from 500 MHz to 8.5 GHz. The conversion from the complex reflection coefficient into the real part (ε’ (ω)) and imaginary part (ε’’ (ω)) of the complex permittivity (ε∗(ω)) was automatically executed by the Keysight software (Keysight N1500A, Santa Rosa, CA, USA). The probe was maintained in a fixed position with a holder under the fume hood in order to minimise measurement errors introduced by potential cable movement. A lift table was used to position the tissue sample in contact with the probe. The temperature of each sample was constantly monitored during measurement using an infrared thermometer.

Calibration was performed at the beginning of each measurement session; it was repeated when drifting of the measurements was observed and every two hours, as required. Measurements were performed on three standard loads: open circuit, short circuit and deionised (DI) water. The mean temperature value of the DI water used for the calibration was 22.0 ± 2.2 °C.

The quality of the calibration was validated with measurements on reference liquid: 0.1 M NaCl solution was measured immediately following the calibration and at the end of the measurement session for validation. The mean temperature value of the 0.1 M NaCl solution used for the validation was 22.6 ± 1.4 °C.

The tissue handling and measurement procedures, included calibration and validation, were conducted under the fume hood, which introduced vibrations linked to the ventilation; however, it ensured a constant and uniform temperature across the samples and the measurements.

### 2.3. Measurement Protocol

On the day of sampling, the lung resection sample was obtained in a fresh unfixed state and immediately transferred to the anatomic pathology laboratory in a sealed container. On receipt, the sample was assigned a histopathology laboratory number, and tissue block cassettes were printed ahead of sampling. The case was assigned to the sampling pathologist for correlation of subsequent histology. The sample was transferred to a fume hood with appropriate ventilation to prevent inhalation of respiratory pathogens from the fresh lung tissue. The pathologist made a limited incision to access the tumour tissue within the resected lung specimen, without impinging upon the bronchial/vascular and stapled margin, and avoiding disruption of the tumoral pleural surface. A second incision was made to access non-neoplastic tissue, typically at least 5 cm from the tumour site.

On exposure of an agreed site, whether this was a neoplastic sample or not, the scientist ensured contact between the dielectric measurement probe and the tissue. Measurements were taken from:

- The tumour;
- An area of non-neoplastic lung tissue.

For each measurement location, five consecutive measurements were acquired in order to reduce measurement error. Finally, the measurement location was marked. The pathologist observed the area carefully and, with a fresh scalpel, took a block of tissue from each of the areas measured, taking note of the measurement code and the cassette code for further data correlation. The cassettes were fixed in 10% neutral-buffered formalin with the specimen in the appropriate container and marked as sampled on the front of the pathology request form. After 24–48 h fixation, the specimens were then sampled as per usual protocol for lung resection specimens, in addition to the cassettes already sampled from that specimen.

**Figure 1:**
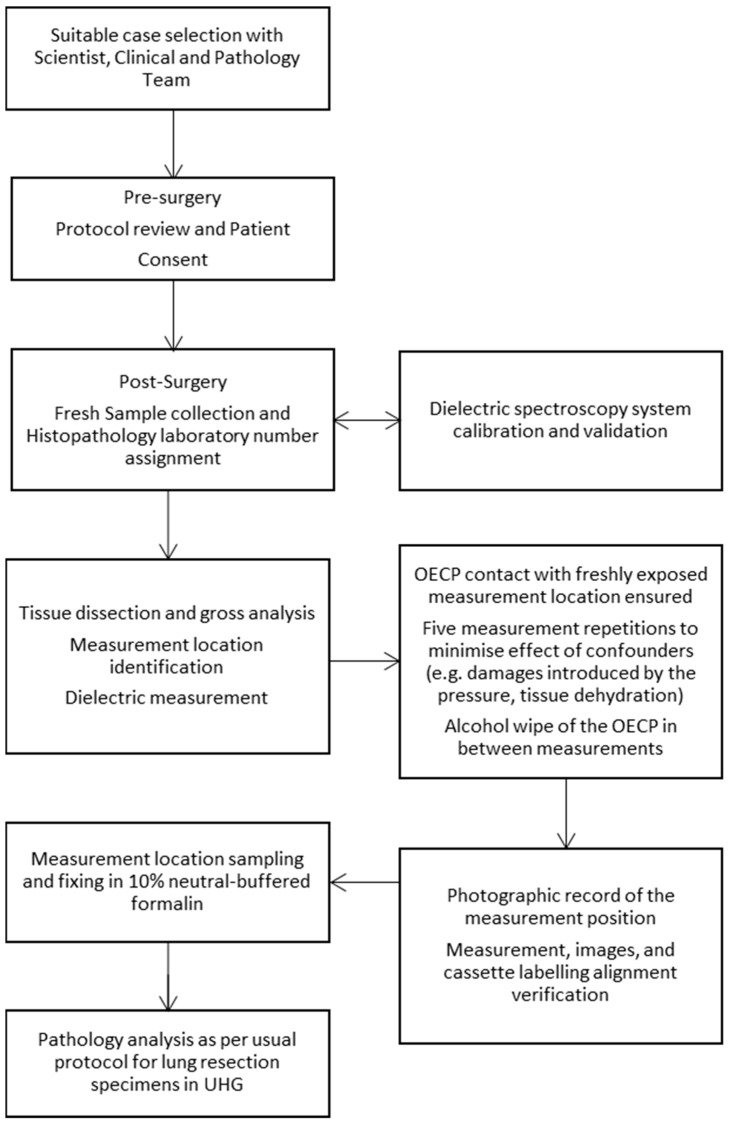
Study protocol and methodology.

The entire study workflow is schematised in Figure 1.

## 3. Results and Discussion

To the best of our knowledge, this study provides, for the first time, a comprehensive evaluation of the dielectric properties of non-cancerous and cancerous human lung tissue samples [24]. The knowledge of these properties at the operating frequency of microwave ablation devices allows the optimisation of the applicator design and improvement in the energy transfer control.

Dielectric property values for lung tissue and different pulmonary tumours, including the most common non-small cell carcinoma (NSCC) such as squamous cell carcinomas and adenocarcinomas, are reported across the microwave frequency range between 500 MHz and 8.5 GHz from surgical resection samples. Dielectric data are correlated with the tissue morphology.

### 3.1. Patient Characteristics

A summary of clinical and pathological characteristics is provided in Table 1. A total of 12 resections, which included 2 pneumectomies and 10 lobectomies are included.

### 3.2. Dielectric Results

The mean temperature value of the measured tissue samples was 19.5 ± 2.4 °C.

Each measurement point was identified and correlated with the results of the morphology review. For each measurement point, five measurements were conducted.

A total of 300 data points were obtained from 60 measurements points defined from non-neoplastic lung samples. These samples included normal and congested parenchyma as well as emphysematous lung and inflamed tissue with patchy fibrosis. Dense fibrotic tissue was also identified but excluded from this count and will be discussed later.

A total of 245 data points were collected over 49 measurements points defined from neoplastic samples. These samples include all the tumours listed in Table 1.

**Figure 2:**
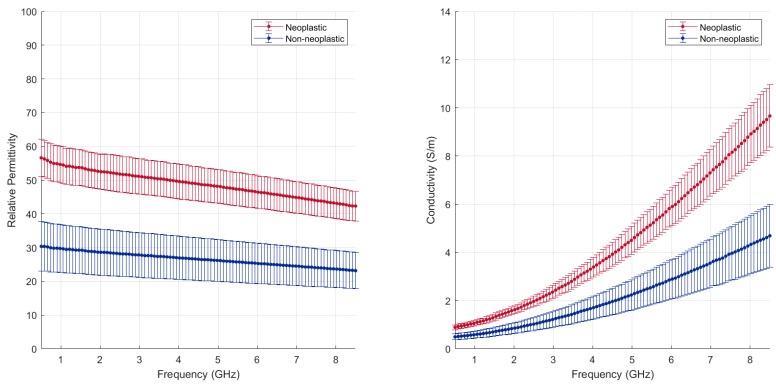
Mean and standard deviation across all the samples collected, independently from the type of tumour or lung conditions (dense fibrosis excluded).

Figure 2 displays all the data collected, (with the exception of dense fibrotic tissue) and reported as a function of the frequency range investigated. Average and standard deviation values are obtained, pooling the non-neoplastic dataset (blue line) and the neoplastic dataset (red line). A sizable difference in the dielectric properties, and thus in the dielectric response, of the normal lung parenchyma and the malignant tissue is shown (*p* < 0.001). The denser structure and the higher water content of the tumour result in dielectric properties about two-fold higher than those observed in the non-neoplastic lung tissue.

The sharp contrast observed between the neoplastic tissue and the parenchyma highlights the possibility of targeting energy selectively in tumours while facilitating functional tissue sparing [25,26]. The higher dielectric properties of the neoplastic tissue favours controlled energy propagation in tumours and suggests higher microwave energy absorption efficiency, thus higher heating efficiency, compared to the non-neoplastic lung tissue.

#### 3.2.1. Non-Neoplastic Lung Tissue

**Figure 3:**
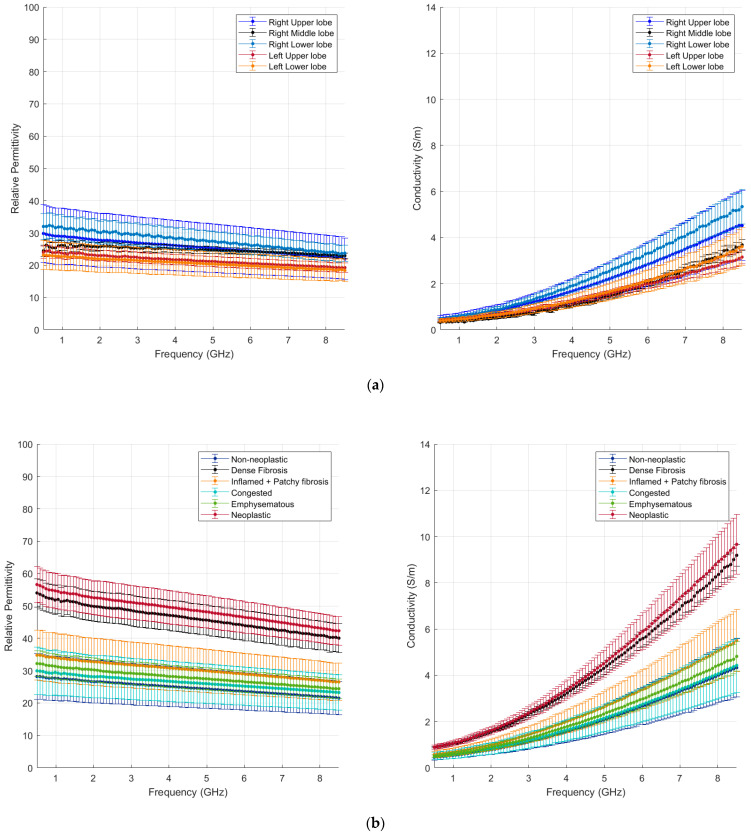
Mean and standard deviation of non-neoplastic lung tissue differentiated by (**a**) lobe and (**b**) condition (dense fibrosis included).

The data collected from locations classified as non-neoplastic after histology are reported in Figure 3a,b as a function of the frequency range investigated. Average and standard deviation values are reported. The lung data are pooled and compared based on their location in the lung (Figure 3a). A wide variability in the data collected can be observed, but there is no significant difference in the lung dielectric properties depending on the lobe investigated (*p* > 0.05).

Figure 3b reports the lung data pooled and compared based on the underlying condition, presented in comparison with the pooled tumour data as well (red line). In particular, the data on dense fibrosis tissue are reported (black line). A total of 15 data points were collected over 3 measurement points for dense fibrosis tissue.

While emphysematous (green line) and congested (light blue line) parenchyma do not show dielectric properties that differ significantly from normal lung tissue, the presence of fibrosis can increase the lung tissue dielectric properties. In the case of inflamed tissue and patchy fibrosis (orange line), the increase observed is not significant with respect to non-neoplastic tissue (*p* > 0.05). Dense fibrosis is significantly different from the remaining non-neoplastic lung tissue (*p* = 0.009) and presents dielectric properties similar to the results from the tumour tissue (*p* > 0.05). Dense fibrosis reduces the air spaces, forming a dense area of connective tissue that leads to higher conductivity, similar to tumour growth. Interestingly, the values found in this study for lung fibrosis match the dielectric values of ex vivo human cirrhotic liver [14].

We conclude that, given a high water content (>70%) and high tissue cellularity, tissue density plays a dominant role in the dielectric properties of lung tissue.

#### 3.2.2. Neoplastic Lung Tissue

**Figure 4:**
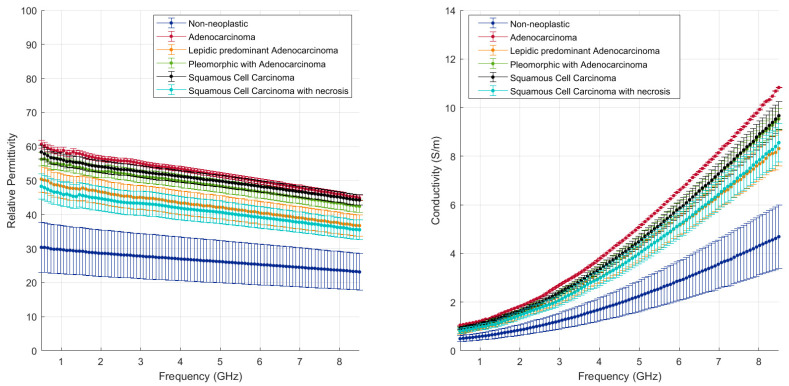
Mean and standard deviation of NSCC tumours differentiated by type against background lung (dense fibrosis excluded); effect of necrosis and lepidic components are highlighted.

**Figure 5:**
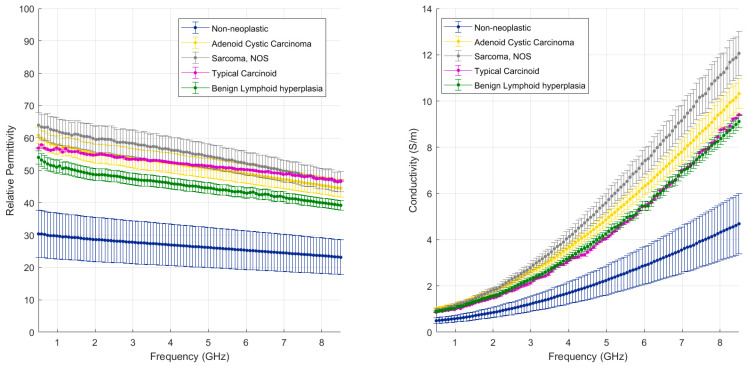
Mean and standard deviation of the other tumours detailed in Table 1 (non-NSCC) differentiated by type against background lung (dense fibrosis excluded).

Figure 4 and Figure 5 illustrate the measured tumour values differentiated by tumour type, as reported in Table 1. Figure 4 reports the dielectric property trends of NSCC tumours as a function of the frequency range investigated. Average and standard deviation values are reported. Data collected from adenocarcinomas and squamous cell carcinomas are pooled and reported; necrosis and lepidic components are highlighted. Figure 5 reports the remaining tumour data.

Across the different neoplastic tissue samples, different dielectric behaviours can be observed in Figure 4 and Figure 5. Among the NSCC, squamous cell carcinoma (black line) and adenocarcinoma (red line) show little data variability, except when associated with necrotic (light blue line) or lepidic (orange line) components. Lepidic adenocarcinomas preserve the alveolar structures of the lung; thus air inclusions are still present and visible. The presence of air inclusion, characterised by lower dielectric property values, determine the higher data variability and the lower observed trends. For necrotic tissue, a less dense structure with few to no viable cells and a majority of necrotic debris can be observed, resulting in lower water content, lower cytoplasm content and broken nuclei.

Interestingly, the values found in this study for NSCC lung tumours, in the absence of necrotic or lepidic components, match results reported for hepatocellular carcinomas [14]. Thus, adenocarcinomas across different organs or primary origin appear to show similar dielectric response at microwave frequency.

Among the other tumours, the least data variability is observed in typical carcinoid (purple line), which shows a different relaxation trend for dielectric permittivity over frequency from the other tumours. The proliferation/growth rate of carcinoids is generally lower than NSCC; carcinoid tumours are composed of neuroendocrine cells, which differ from the epithelium cells from which NSCC is derived. These differences may account for the variable results observed.

The remaining malignant tumours show similar relaxation trends to the NSCC tumours and high data variability. The adenoid cystic carcinoma (yellow line) presents values in the range of those reported for the NSCC. The sarcoma (grey line) shows the highest values of relative permittivity and conductivity measured in this study. The sarcoma in this study is composed of spindled cells in a fascicular architecture, and it is a cellular dense tumour with few air pockets. It may have different cellular and dielectric properties linked to differences in the water content and ultrastructure of the cells due to the mesenchymal rather than epithelial nature of the cells. Indeed, cells undergoing epithelial to mesenchymal transition experience a change in polarity, cytoskeleton structure and cell-to-cell adhesion structure [27].

The nodular lymphoid hyperplasia sample shows slightly lower values of permittivity, close to the values of NSCC with lepidic or necrotic components, and is thus within the range of the other malignant tumours. The level of cellularity and density is built up by lymphocyte cells and fibrosis modulating the lung architecture.

These observations point to the dominant role the tissue density, or gaseous volume, plays in the dielectric properties of lung tissue.

The dielectric data collected for each measurement point are detailed together with their morphological analysis in Appendix A (Table A1 and Figure A1, Figure A2, Figure A3, Figure A4, Figure A5, Figure A6, Figure A7, Figure A8, Figure A9, Figure A10, Figure A11 and Figure A12). In the following section, two NSCCs are compared to better discuss the correlation between dielectric measurements and morphological analysis.

#### 3.2.3. NSCC Case Study

A poorly differentiated squamous cell carcinoma and a well-differentiated lepidic adenocarcinoma are selected for comparison. Table 2 reports the dielectric data of the selected samples and correlates with the morphological features. The two cases are directly compared.

The influence of the tissue morphology of the two selected tumours on the dielectric data can be observed as follows:

- In the poorly differentiated squamous cell carcinoma, the lung morphology is lost, a denser structure is observed, and higher dielectric properties characterised by less variability are reported;
- In the well-differentiated adenocarcinoma with a lepidic structure, a less dense structure resembling the lung morphology can be observed, and higher data variability with average values lower than the comparison case is reported.

Comparing the dielectric data with the tissue morphology, in Table 2, as well as in the Appendix A, a correlation between the two features is confirmed as follows:

- In the neoplastic tissue, where the lung morphology is impaired and fewer or no air gaps are present, higher dielectric properties are observed, close to tissues richer in water content, e.g., liver;
- In the non-neoplastic tissue, where the morphology structure is less dense and air pockets are present, lower dielectric properties are observed.

#### 3.2.4. Microwave ablation operating frequency

In Table 3, the values of relative permittivity and effective conductivity measured for each tissue type at 2.45 GHz are listed as average and standard deviation.

The overall average values at 2.45 GHz for the neoplastic lung tissue are 52.0 ± 5.4 for relative permittivity and 1.9 ± 0.2 S/m for effective conductivity, compared to the non-neoplastic lung tissue (excluding fibrous tissue) with values of 28.3 ± 6.7 for relative permittivity and 1.0 ± 0.3 S/m for effective conductivity. The dielectric properties of the tumours are approximately two-fold the values found for non-neoplastic parenchyma.

Lung cancer is often present in fibrotic or emphysematous lungs [28,29]. The data from Table 3 confirm that, in presence of emphysematous parenchyma or patchy fibrosis, the dielectric pulmonary response does not differ from the normal lung one, while in the presence of dense fibrosis no significant dielectric difference can be observed compared with neoplastic tissue, as highlighted in Figure 3b. Ground-glass-dominant nodules (GGN), such as lepidic adenocarcinomas and benign tumours, are no exception: their properties are not significantly different from lung dense fibrosis, but they are distinguishable from patchy fibrosis or emphysematous parenchyma.

The data reported in this study were collected from resected samples, i.e., ex vivo. Ex vivo measurements allow for more accurate control of the measurement set-up [13]. In vivo dielectric measurements are largely affected by difficult-to-control confounders introduced by living tissue and the additional limitations introduced by studies performed in human subjects such as patient safety and ethical considerations. Investigations conducted in more easily controllable animal studies suggest that in vivo dielectric properties can be expected to be about 10% higher than those found in ex vivo environments due to the presence of active perfusion in the tissue [21]. Moreover, the measured samples were at room temperature and should be considered as non-inflated, yet they can be considered representative of the physiological thermal state during active breathing (inflated). Dielectric properties are not expected to significantly change in physiological temperatures. Only when hyperthermic damage starts to occur can significant variations be observed [30,31]. As reported in [19] for ovine ex vivo lungs, dielectric properties tend to decrease with increasing air content (inflated lungs), but no significant difference is reported. Thus, similarly, ex vivo human inflated lungs are expected to be characterised by dielectric properties in the lower range of the values reported in this work.

## 4. Conclusions

In this study, we presented the first histology-validated assessment of human lung ex vivo dielectric properties. Values are reported for neoplastic and non-neoplastic lung tissue in the frequency range of interest for microwave ablation applications. Tissue density, cellularity and gaseous content are shown to have a dominant role in the resulting dielectric properties of the tissue. Neoplastic tissue, as well as dense fibrosis, showed values higher than those of normal lung tissue, including inflamed, congested and emphysematous. Lung tumours are up to two times more dissipative than lung parenchyma. This observation is favourable for microwave thermal ablation therapies for the following reasons:

- Microwave ablation applicators are historically designed to better operate in highly dissipative materials, such as liver or kidney tissue;
- The dissipative nature of tissue characterised by higher dielectric properties facilitates the absorption of the electromagnetic field radiated from the applicator in the tissue and thus the subsequent increase in temperature;
- Such considerations can have a significant impact on the design of minimally invasive microwave ablation applicators and treatment planning tools, and should be taken into account to deliver more precise and efficient ablation tools.

## Figures and Tables

**Table 1:** Clinicopathological Features of Patients and Specimens, *n* = 12.

Median Age, Years (Range)	70 (55–83)
Sex, no. (%)	
Men	5 (42)
Women	7 (58)
Smoking status, no. (%)	
Current smoker	1 (8)
Former smoker	7 (58)
Never smoker	4 (33)
Unknown	- (-)
Histology, no. (%)	
NSCC	8 (67)
Adenocarcinoma	3 (25)
Squamous cell carcinoma	5 (42)
Other tumours	4 (33)
Sarcoma, NOS	1 (8)
Adenoid cystic carcinoma	1 (8)
Typical carcinoid	1 (8)
Nodular lymphoid hyperplasia	1 (8)
Type of specimen, no. (%)	
Resection	
Pneumonectomy	2 (17)
Lobectomy	10 (83)
Sector resected	
Upper Right Lobe	4 (33)
Middle Right Lobe	1 (8)
Lower Right Lobe	2 (17)
Upper Left Lobe	2 (17)
Lower Left Lobe	1 (8)
Right Lung	1 (8)
Left Lung	1 (8)
Stage *, no. (%)	
I	2 (17)
II	6 (50)
>III	2 (17)
Unknown/Not Applicable	2 (17)

* Staging by TNM 8th ed. NSCC: non-small cell carcinoma; NOS: not otherwise specified.

**Table 2:** Gross appearance, morphology and dielectric properties comparison of two NSCC.

	Poorly Differentiated Squamous Cell Carcinoma	Well-Differentiated Lepidic Adenocarcinoma
Gross appearance	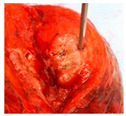	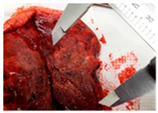
Morphology	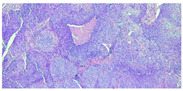 Tumour centre *50*× H&E (M5: m.p.1, m.p.2)	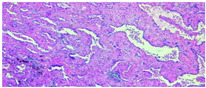 Tumour centre *20×* H&E (A1: m.p.1, m.p.2, m.p.3)
	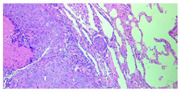 Tumour margin *20×* H&E (M6: m.p.1, m.p.2)	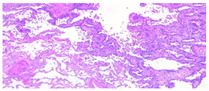 Tumour margin *20×* H&E (A2: m.p.1, m.p.2, m.p.3, m.p.4)
Relative Permittivity	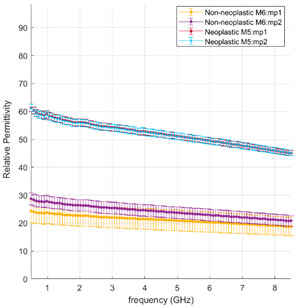	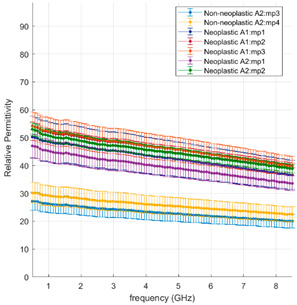
Effective Conductivity	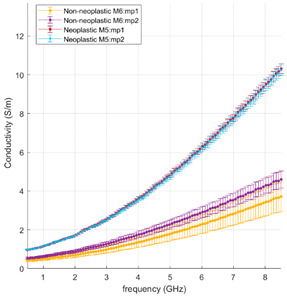	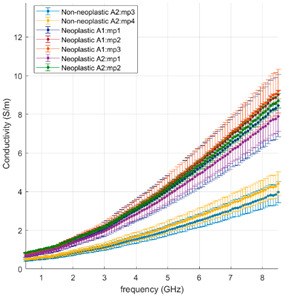

m.p.: “measurement point” progressive measurement point number; A1, A2, M5, M6: cassette number.

**Table 3:** Dielectric values of lung tissue and lung tumours at 2.45 GHz.

	Relative Permittivity	Effective Conductivity (S/m)
Lung tissue		
Normal (including Inflamed, congested and emphysematous)	28.3 (6.7)	1.00 (0.26)
Dense Fibrosis	49.3 (4.6)	1.83 (0.10)
NSCLC		
Adenocarcinoma	55.9 (0.8)	2.13 (0.02)
Pleomorphic	52.2 (1.8)	1.90 (0.09)
Lepidic pattern	45.8 (3.5)	1.72 (0.12)
Squamous cell carcinoma	53.3 (1.7)	1.92 (0.10)
With necrosis	43.8 (3.5)	1.71 (0.19)
Other tumours		
Sarcoma, NOS	59.5 (4.2)	2.22 (0.14)
Adenoid cystic carcinoma	54.7 (2.9)	2.09 (0.09)
Typical carcinoid	54.6 (0.2)	1.77 (0.03)
Nodular lymphoid hyperplasia	48.3 (1.8)	1.84 (0.07)

NOS: not otherwise specified.

**Figure A1:**
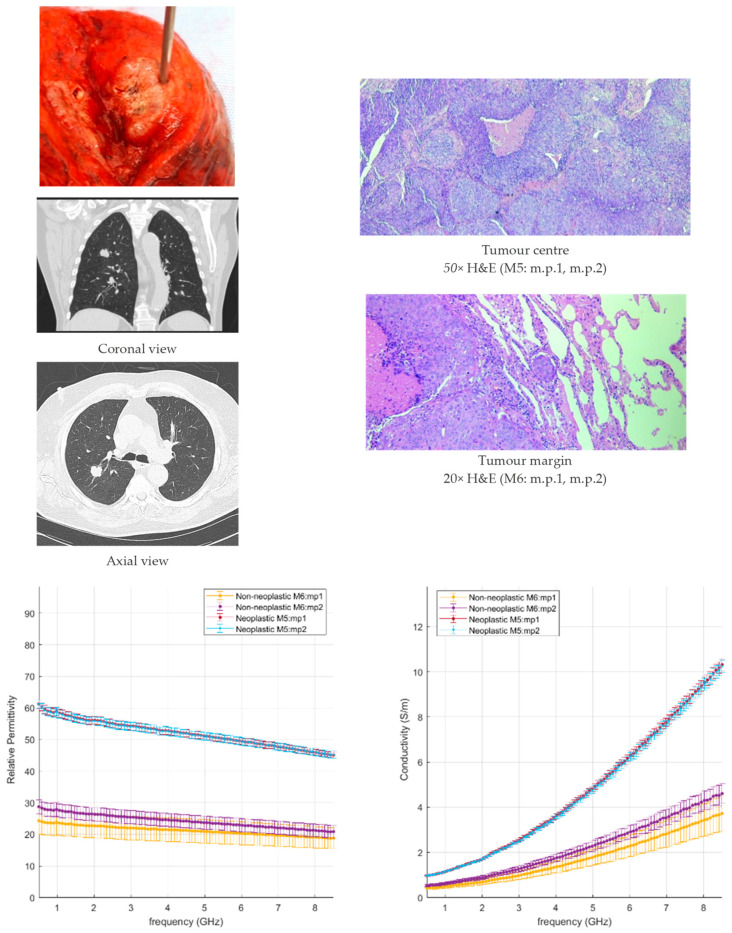
Poorly differentiated squamous cell carcinoma (22 × 19 × 26 mm), right upper lobe.

**Figure A2:**
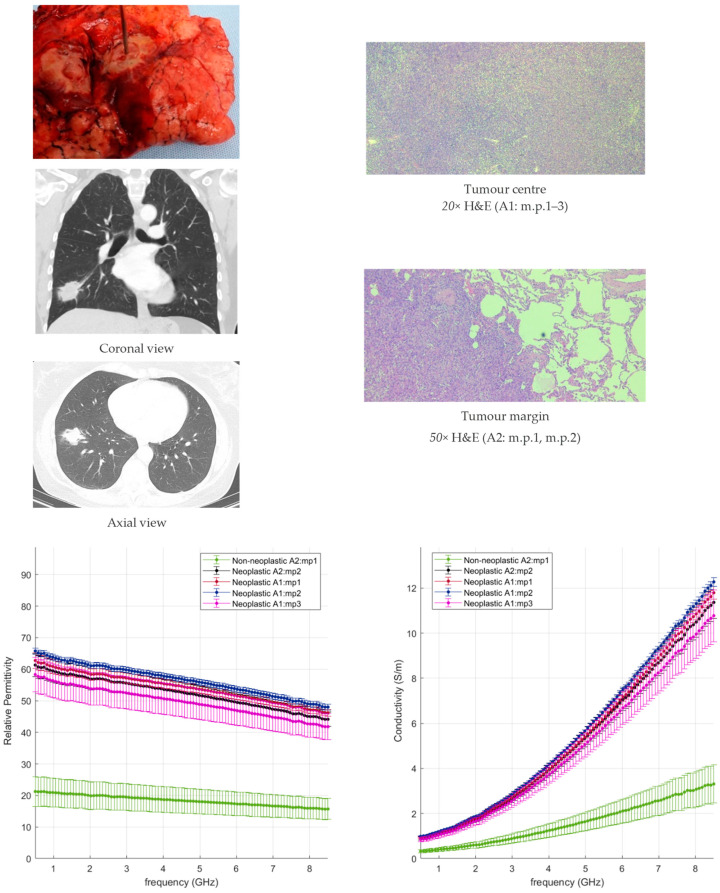
Sarcoma, NOS (35 × 28 × 22 mm), right lower lobe.

**Figure A3:**
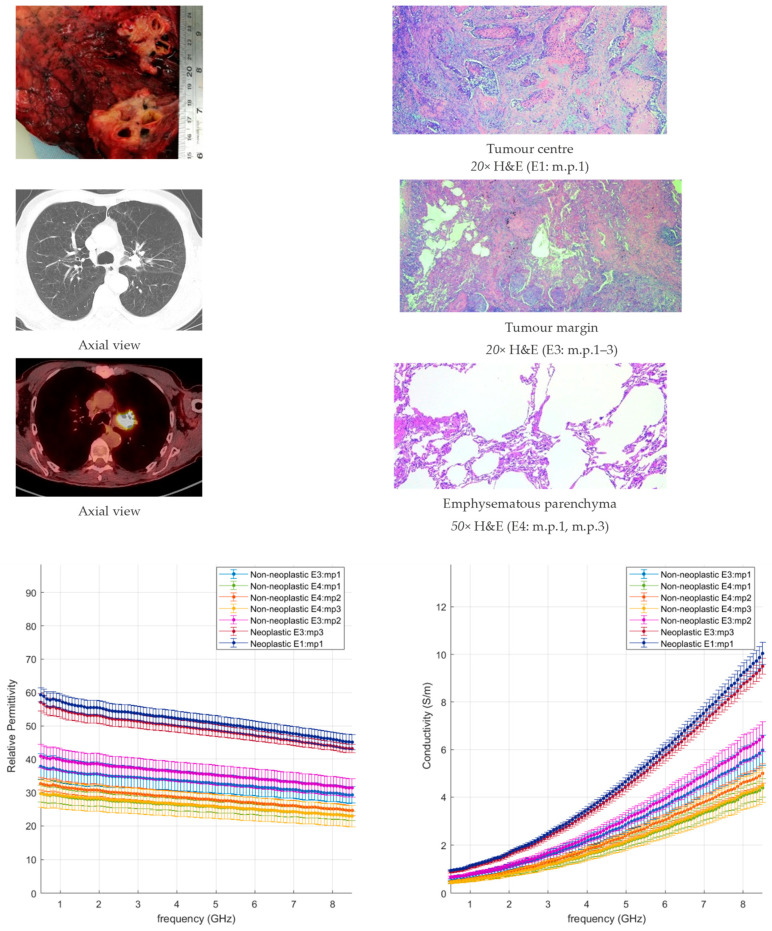
Well-differentiated squamous carcinoma with desmoplastic reaction, fibrotic connecting tissue and tumour nests with keratin and necrosis inside (45 × 40 × 25 mm), left lung main bronchus.

**Figure A4:**
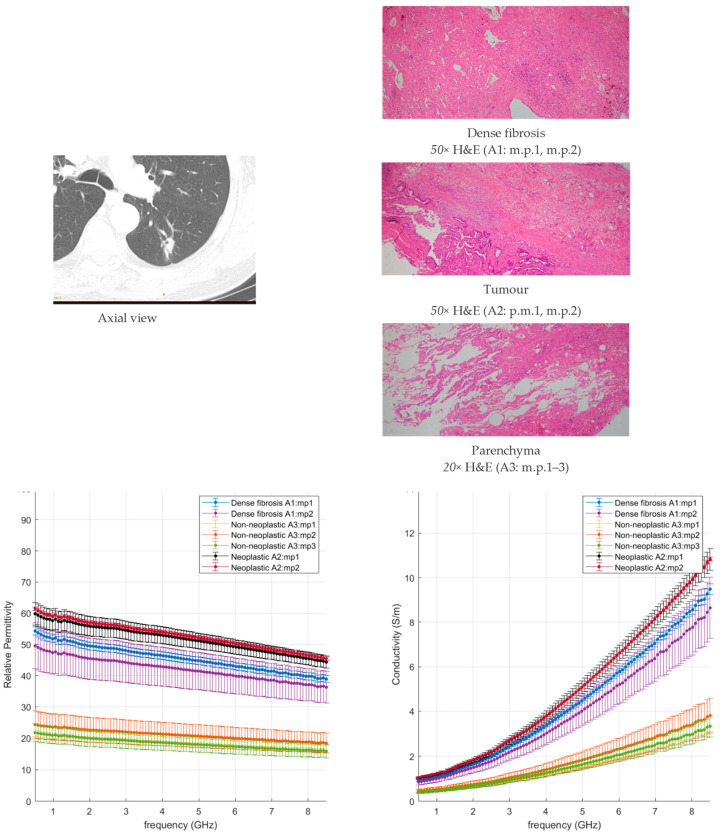
Adenocarcinoma (20 × 17 × 10 mm), left lower lobe.

**Figure A5:**
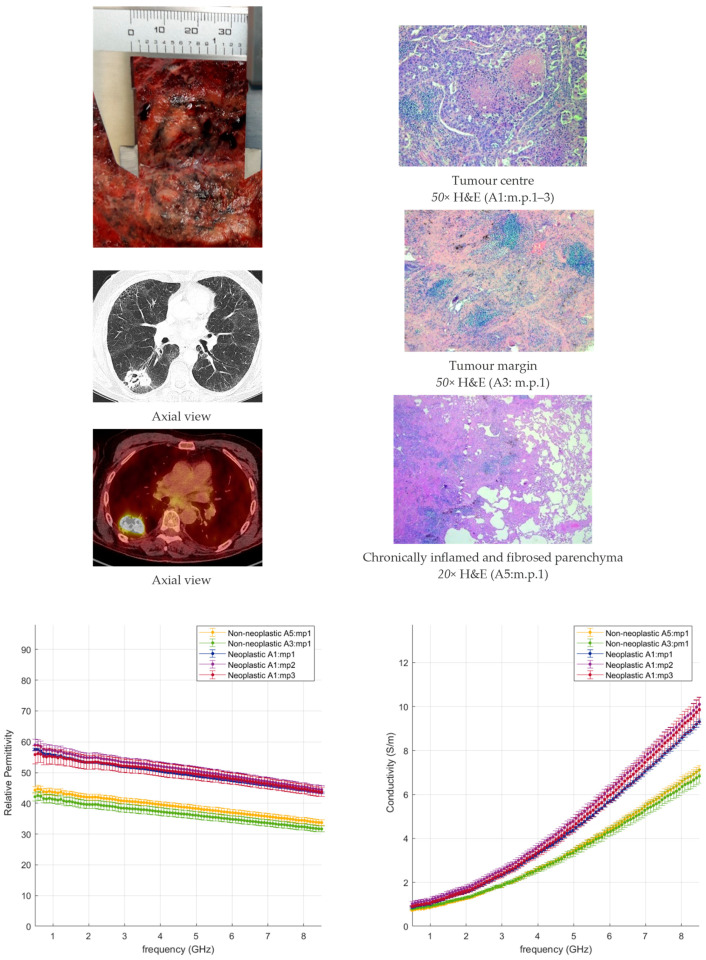
Pleomorphic carcinoma with adenocarcinoma (45 × 30 × 35 mm), right lower lobe.

**Figure A6:**
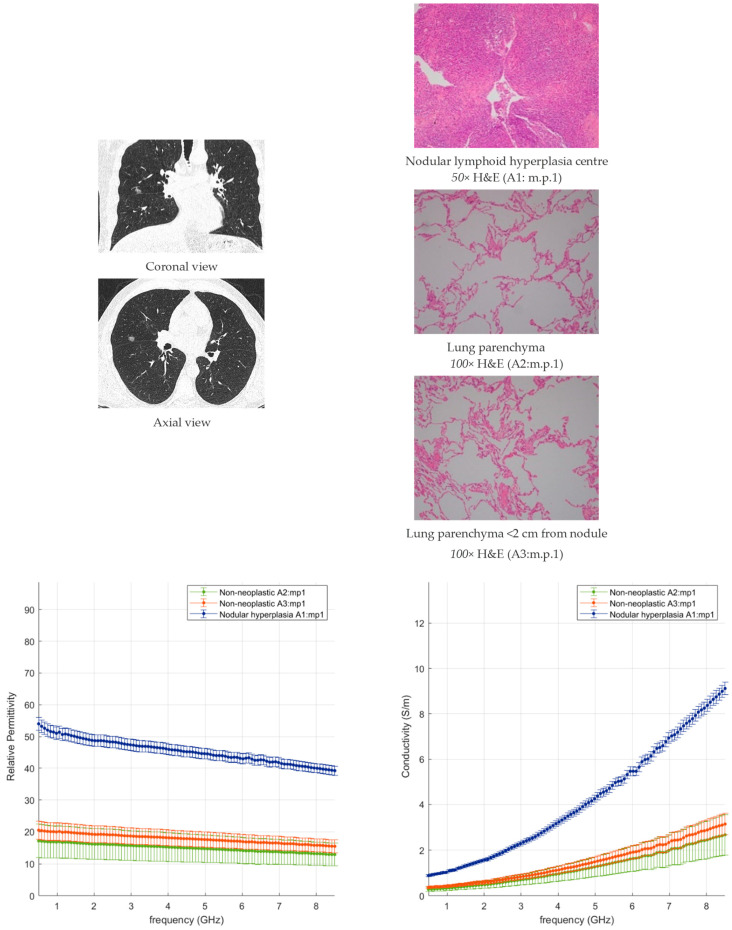
Nodular lymphoid hyperplasia (10 mm in diameter), right upper lobe.

**Figure A7:**
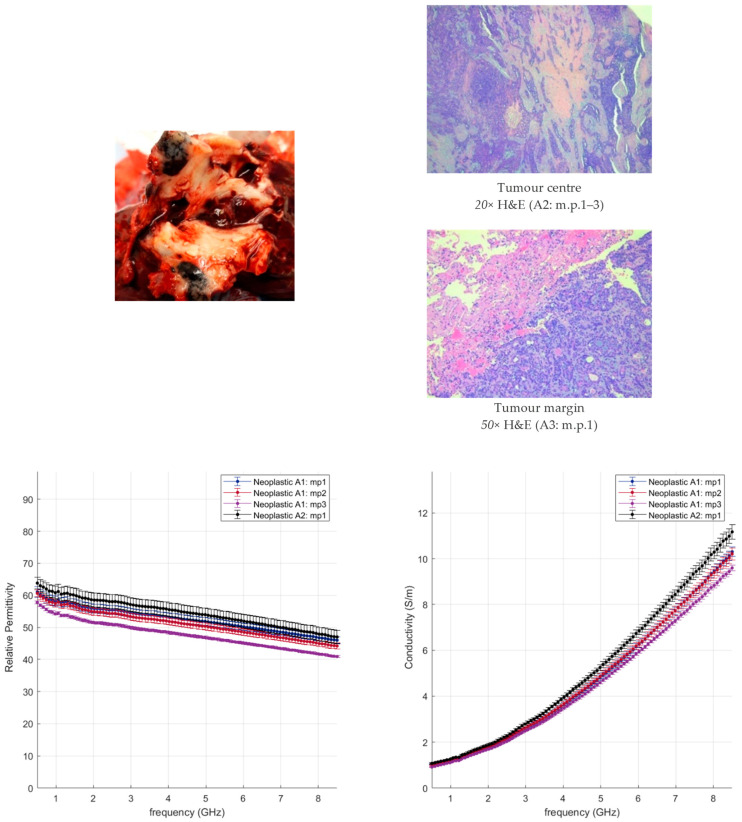
Adenoid cystic carcinoma invades into cartilage and focally ulcerates the main bronchial lining (36 × 23 × 18 mm), right lung main bronchus.

**Figure A8:**
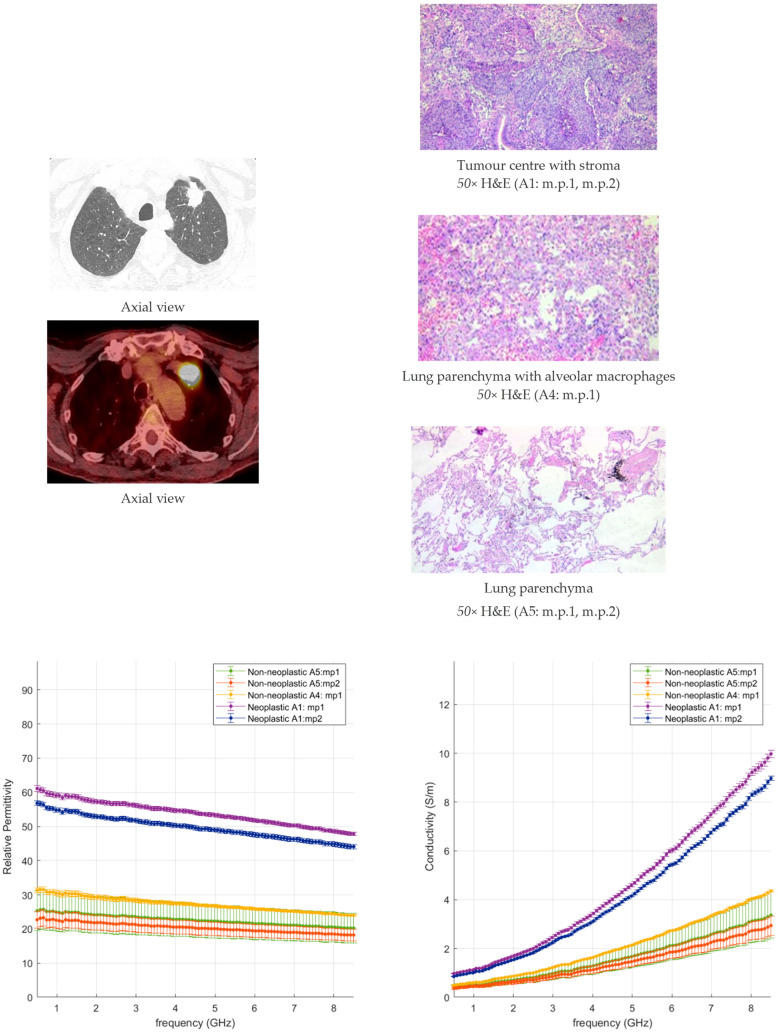
Moderately to poorly differentiated focally keratinising squamous cell carcinoma with a nested architecture and frequent areas of necrosis (38 × 38 × 25 mm), apex left upper lobe.

**Figure A9:**
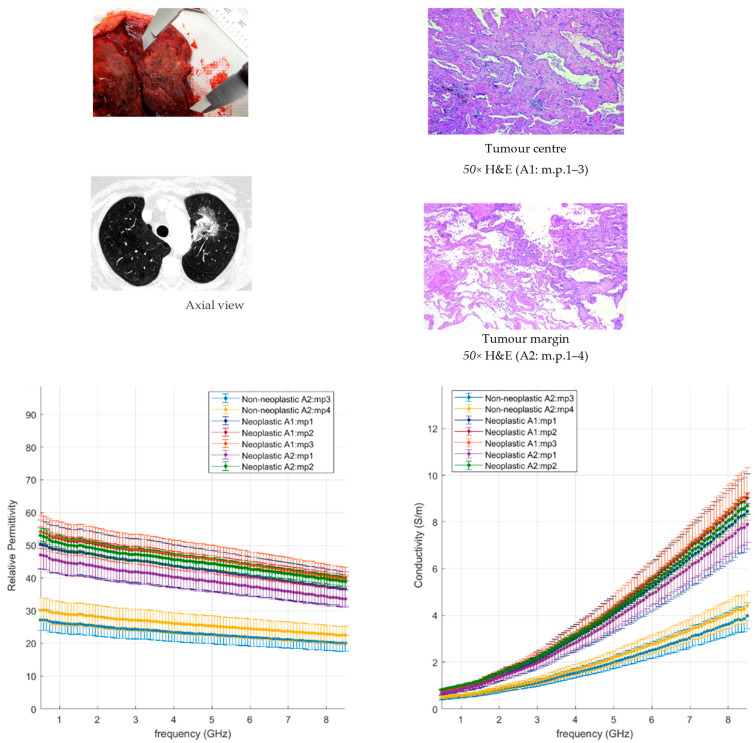
Adenocarcinoma with a predominantly lepidic pattern of growth (65%), with papillary (30%) and focal acinar (5%) areas (50 × 35 × 30 mm), left upper lobe.

**Figure A10:**
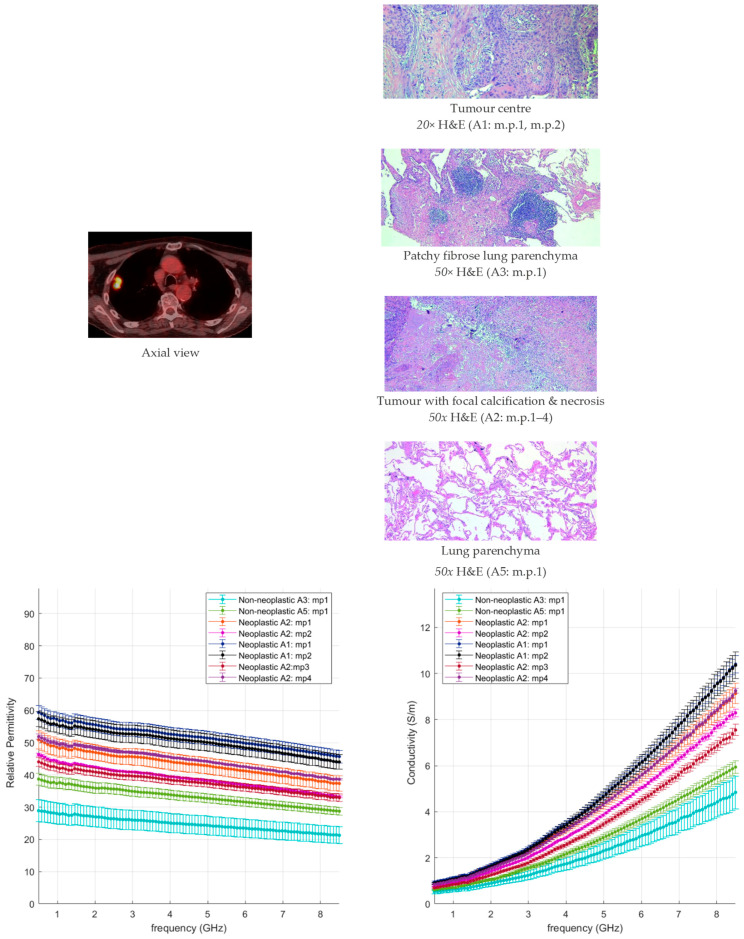
Moderately differentiated keratinising squamous cell carcinoma with focal necrosis and dystrophic calcification (38 × 18 × 20 mm), right upper lobe.

**Figure A11:**
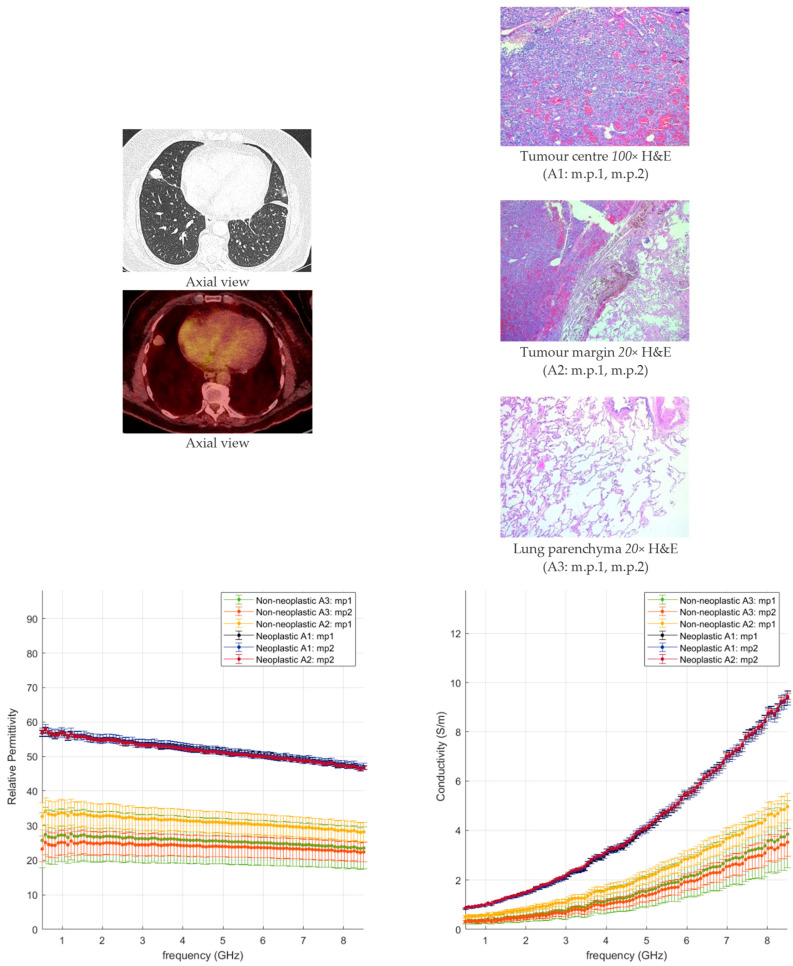
Typical carcinoid well-circumscribed lesion composed of trabecular areas, rosette-like structures and occasional solid foci, with medium-sized oval/epithelioid nuclei with disperse chromatin, and some visible nucleoli (21 × 15 × 12 mm). Adjacent to the tumour there are multiple foci of neuroendocrine hyperplasia, forming small carcinoid tumourlets (approx. 10 in sections examined). A large tumourlet with spindled growth adjacent to tumour. Background lung is heavily congested, with multiple pigmented macrophages. Right middle lobe, focally extends to the pleural surface.

**Figure A12:**
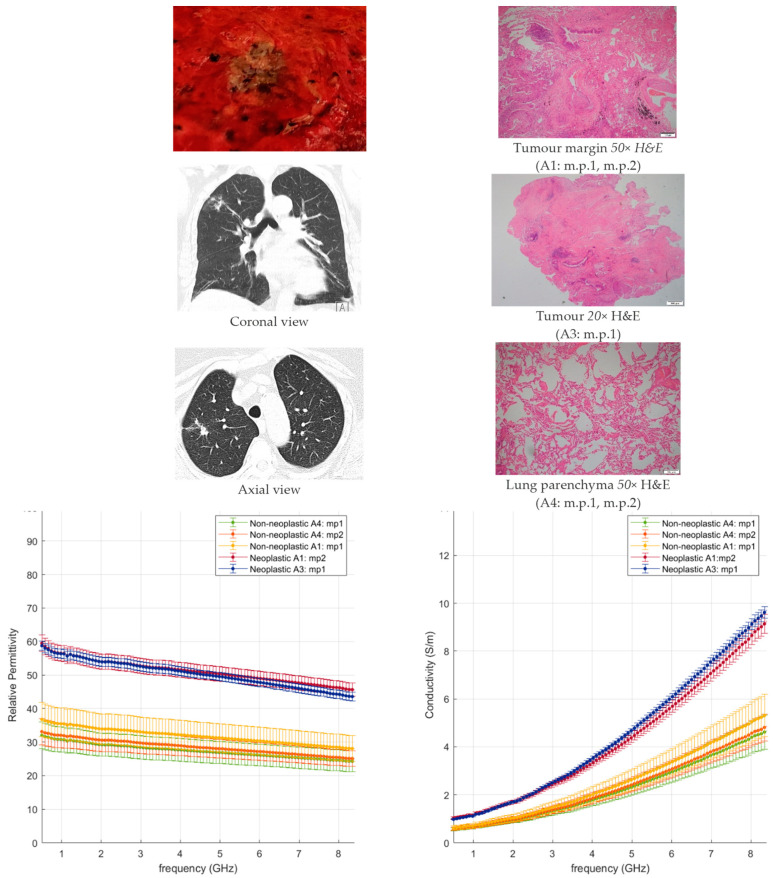
Invasive moderately differentiated squamous cell carcinoma (20 × 17 × 15 mm), right upper lobe.

## Data Availability

Data are contained within the article.

## Appendix A

In the following, the gross images (where available), CT/PET images (where available), dielectric data and morphology assessments resulting from this study are reported in detail. For each sample, a gross visualisation of the neoplastic resection is reported. The histology images presented contain the measurement points listed in the dielectric property plots.

The information for each sample is reported in Table A1 and linked with the respective Figure A1, Figure A2, Figure A3, Figure A4, Figure A5, Figure A6, Figure A7, Figure A8, Figure A9, Figure A10, Figure A11 and Figure A12.

**Table A1:** Sample information.

Figure	Histology	Site of Specimen	Size of Tumour	Pathology Stage	Smoking Status	Sex	Age	Cassette Samples (A-, E-, M-)	Measurement Points (m.p.)
**A1**	Squamous carcinoma	Right upper lobe	26 mm	pT1c	Ex-smoker	M	83	4	7
**A2**	Sarcoma, NOS	Right lower lobe	35 mm	N/A	Ex-smoker	F	66	5	11
**A3**	Squamous carcinoma	Left lung	45 mm	pT3	Ex-smoker	M	73	5	10
**A4**	Adenocarcinoma	Left lower lobe	22 mm	pT2	Ex-smoker	M	74	6	10
**A5**	Pleomorphic carcinoma with adenocarcinoma	Right lower lobe	45 mm	pT2b	Smoker	M	68	5	9
**A6**	Nodular lymphoid hyperplasia	Right upper lobe	10 mm	N/A	Never smoked	M	55	6	6
**A7**	Adenoid cystic carcinoma	Right lung	42 mm	pT3	Never smoked	F	60	2	4
**A8**	Squamous carcinoma	Left upper lobe	38 mm	pT2a	Ex-smoker	M	78	6	11
**A9**	Adenocarcinoma (lepidic-predominant)	Left upper lobe	50 mm	pT2b	Non-smoker	F	81	4	14
**A10**	Squamous carcinoma	Right upper lobe	38 mm	pT2a	Ex-smoker	M	76	5	14
**A11**	Typical carcinoid	Right middle lobe	21 mm	pT2	Never smoked	F	63	3	6
**A12**	Squamous carcinoma	Right upper lobe	20 mm	pT1b	Ex-smoker	F	72	5	9
